# BIONDA: a free database for a fast information on published biomarkers

**DOI:** 10.1093/bioadv/vbab015

**Published:** 2021-08-18

**Authors:** Michael Turewicz, Anika Frericks-Zipper, Markus Stepath, Karin Schork, Spoorti Ramesh, Katrin Marcus, Martin Eisenacher

**Affiliations:** 1 Medizinisches Proteom-Center, Ruhr University Bochum, Bochum 44801, Germany; 2 Center for Protein Diagnostics (PRODI), Medical Proteome Analysis, Ruhr University Bochum, Bochum 44801, Germany

## Abstract

**Summary:**

Because of the steadily increasing and already manually unmanageable total number of biomarker-related articles in biomedical research, there is a need for intelligent systems that extract all relevant information from biomedical texts and provide it as structured information to researchers in a user-friendly way. To address this, BIONDA was implemented as a free text mining-based online database for molecular biomarkers including genes, proteins and miRNAs and for all kinds of diseases. The contained structured information on published biomarkers is extracted automatically from Europe PMC publication abstracts and high-quality sources like UniProt and Disease Ontology. This allows frequent content updates.

**Availability and implementation:**

BIONDA is freely accessible via a user-friendly web application at http://bionda.mpc.ruhr-uni-bochum.de. The current BIONDA code is available at GitHub via https://github.com/mpc-bioinformatics/bionda.

**Supplementary information:**

Supplementary data are available at *Bioinformatics Advances* online.

## 1 Introduction

Molecular biomarkers including variations or expression patterns of genes, proteins and miRNAs are indicators for a specific biological process, condition or state including diseases. Knowing all biomarkers published to date for the target disease is crucial for researchers at various steps of e.g. biomarker studies. However, the number of biomarker-related articles increases dramatically (∼50 000 new articles in 2019) and it becomes infeasible for individual researchers to read all of them. Thus, there is a need for an intelligent system that extracts the currently available knowledge from biomedical texts and provides it as structured information including the biomarker–disease relations in a user-friendly way.

However, most biomarker databases are not generally applicable. For example, the disease-specific OncoMX (Dingerdissen *et al.*, 2020), CIViCmine (Lever *et al.*, 2019) and ResMarkerDB (Pérez-Granado *et al.*, 2019) for cancer and the biomarker-specific OMIM (Amberger *et al.*, 2019; genes and genetic disorders) provide no general view on biomarkers and diseases. Some databases like GOBIOM (https://gobiomdbplus.com, 11 July 2021, date last accessed) are not free and/or require a registration. There are databases like Disease-related Biomarker Database (Bravo *et al.*, 2014), which are not regularly maintained. MarkerDB (Wishart *et al.*, 2021) is a free and basically general resource. However, it has a different focus as it concentrates on already well-studied biomarkers including only 143 protein biomarkers, 643 diseases, no miRNAs and no biomarker candidates.

In contrast, BIONDA (BIOmarker and biomarker caNdidates DAtabase) provides structured information on all gene, protein and miRNA biomarkers and biomarker candidates mentioned in scientific article abstracts. This information is automatically acquired from high-quality source databases and updated monthly using text mining. BIONDA is not limited to particular diseases, is freely accessible without registration via a user-friendly web application and its database entries are rated by a score for biomarker reliability.

## 2 Implementation and content

**Fig. 1.**
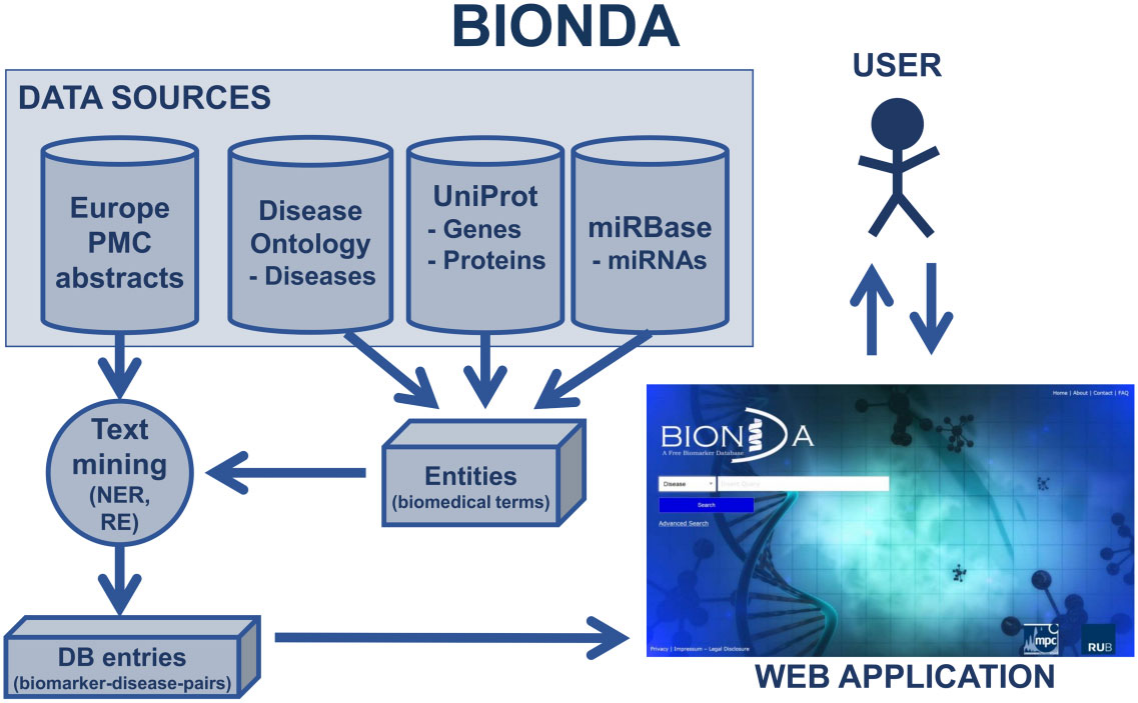
Architecture of BIONDA. BIONDA’s source databases, the text mining-based information acquisition and the homepage of the web application are shown. I have added this.

The basic architecture of BIONDA is shown in Figure 1. As source databases for the raw information, the online resources Europe PMC (https://europepmc.org, 11 July 2021, date last accessed), Disease Ontology (Schriml *et al.*, 2019), UniProt (UniProt Consortium, 2021) and miRBase (Kozomara *et al.*, 2019) are used. All of them are free and renowned databases, which are regularly updated. Information acquisition via text mining is performed as follows:

1. *Information retrieval:* In text mining, information retrieval deals with obtaining or fetching the relevant information from a larger information source. For BIONDA, the relevant information from Europe PMC is automatically imported into our text mining pipeline using the Europe PMC API with the search terms ‘biomarker’ and ‘biological marker’ to filter for biomarker-related publications.
2. *Abstract preprocessing:* The pre-selected abstracts are further processed. Here, the metadata such as authors, year of publication, etc. are extracted and stored separately for later assignment to identified biomarker-disease pairs. Then, the actual abstract texts are pre-processed, including e.g. removal of special characters.
3. *Named entity recognition (NER), named entity normalization (NEN) and relation extraction (RE):* For automatic NER of proteins/genes, miRNAs and diseases in abstracts a dictionary-based approach (Egorov *et al.*, 2004) is employed. For this, Disease Ontology, UniProt and miRBase terms are used as dictionaries. The dictionary approach also inherently performs NEN to unique identifiers for synonyms of recognized terms. RE is based on BIONDA’s biomarker definition, where a biomarker–disease pair is included as a new ‘biomarker’ when at least one co-occurrence of the respective biomolecule and disease in the same abstract sentence was found in Europe PMC.
4. *Statistical scoring:* For each database entry, i.e. biomarker–disease pair, a score is calculated to assess its reliability. The score is a *P*-value computed by the χ^2^ test, applied to the contingency table of the number of co-occurrences of the respective biomarker-disease pair and the occurrences of only the disease or the biomarker.
5. *Storage and updates:* BIONDA stores monthly updated biomarker–disease pairs, re-calculated scores and current information on all associated publications from 1960 until now in a relational database. Previous versions are available and can be provided upon request.

### 2.1 Web application

BIONDA’s web application provides both fast access to the data via a straightforward search field on the homepage and highly specific searches based on publication metadata (e.g. authors, journals, publication years) via an advanced search page. Search results are displayed in a results table providing matching biomarker–disease pairs including their respective scores. The results can be sorted, filtered and downloaded as spreadsheet files. Further features include a community curation module for manual confirmation of correct biomarker–disease pairs (only after log-in via ORCID to ensure expert status) and reporting of incorrect or missing entries. The manual entry confirmation results in a star rating ranging from bronze to gold.

### 2.2 Content

Currently, over 800 000 pre-selected Europe PMC abstracts are digested each month. BIONDA contains above 520 791 biomarker–disease publication tuples, of which 122 592 are unique biomarker–disease pairs. About 34 257 terms from the dictionaries are stored and used (11 865 diseases, 20 408 genes/proteins and 1984 miRNAs). Additionally, due to the COVID-19 pandemic, BIONDA currently provides 18 combinations of genes/proteins from 11 different publications associated with COVID-19 and considered as possible biomarkers, where 12 of these are unique.

## 3 Conclusion and outlook

BIONDA is a text mining-based online resource containing structured and up-to-date information on published molecular biomarkers and biomarker candidates. Its renowned source databases ensure a high quality of included raw data. An evaluation and comparison of BIONDA with MarkerDB and OMIM are shown in the Supplementary Material. As can be seen in Supplementary Tables S2 and S4, BIONDA’s NER (precision: 0.9317, recall: 0.9569, F1 score: 0.9441) and RE (precision: 0.8318, recall: 0.9289, F1 score: 0.8777) outperform MarkerDB and OMIM regarding recall and F1 score. On the other hand, BIONDA is outperformed by the other databases regarding precision. So, BIONDA is focused more on higher recall, while the other databases are focused more on higher precision. Due to its score rating biomarker–disease pairs and community-based manual curation, BIONDA is a reliable and valuable knowledge resource supporting biomedical (literature) research.

BIONDA will be further developed by including full-text articles and preprints as additional data sources and further molecular biomarkers (e.g. metabolites or lipids). Moreover, the text mining pipeline will be constantly optimized to improve precision and recall of the identified biomarker–disease pairs. For example, the usage of modern deep learning-based methods employing transformer and Long Short-Term Memory (LSTM) architectures (Lee *et al.*, 2020) can improve the NER (Digan *et al.*, 2021). However, BIONDA’s web application will remain largely unchanged, so that backend improvements will not affect its usability.

## Software and data availability

The data underlying this article are available in the article and in its online supplementary material.

## Funding

This work was supported by de. NBI, a project of the German Federal Ministry of Education and Research (BMBF) [grant number FKZ 031 A 534A] and Center for Protein Diagnostics (PRODI), a grant of the Ministry of Innovation, Science and Research of North-Rhine Westphalia, Germany.

*Conflict of Interest*: none declared.

## Supplementary Material

vbab015_Supplementary_Data
