# Supplementary material for: BIONDA: a free database for a fast information on published biomarkers
Source: Bioinform Adv. 2021 Aug 18;1(1):vbab015. doi: 10.1093/bioadv/vbab015 (PMC9710600; doi:10.1093/bioadv/vbab015)
Supplement: vbab015_Supplementary_Data [file vbab015_supplementary_data.zip › supplementary file 1.docx]

***Supplementary file 1***

**BIONDA evaluation and comparison with other databases**

# Methods

BIONDA, the BIOmarker and biomarker caNdidates DAtabase (http://bionda.mpc.ruhr-uni-bochum.de), provides information on all gene, protein and miRNA biomarkers and biomarker candidates mentioned in article abstracts. This information is automatically acquired from high-quality source databases and updated monthly using text mining.

In order to assess the reliability of the BIONDA’s content and its automatic text mining information retrieval approach, a manual evaluation has been performed independently by two curators. The here described final evaluation results are the consensus of the two individual evaluation results and shown in supplementary file 2.

For this evaluation, first, 200 abstract sentences were randomly selected from BIONDA’s database content. Then, the curators reviewed whether diseases and biomolecules (genes, proteins and miRNAs), i.e. biomarker candidates, were tagged correctly and completely. So, the true positive (TP), false positive (FP) and false negative (FN) tagging of BIONDA’s named entity recognition (NER) for each of the 200 sentences was recorded (see Table S1). Analogously, BIONDA’s relation extraction (RE) was evaluated (see Table S3). Furthermore, for each sentence it was assessed whether the biomarker-disease relation that was detected by BIONDA (without at least one detected biomarker-disease relation this sentence would be not included in BIONDA) is correct and whether there are FN relations. Then, for both NER and RE precision, recall and the F1 score were calculated based on the overall TP, FP and FN numbers for the 200 sentences (see Table S2 and Table S4).

In order to compare the obtained evaluation results for BIONDA with the performance of similar databases, the entities and relations manually detected in the 200 sentences were searched in the two free databases MarkerDB (Wishart, et al., 2021) and OMIM (Amberger, et al., 2019). The other databases mentioned in the main text were not compared with BIONDA because they are currently not available (Disease-related Biomarker Database (Bravo, et al., 2014)), not free (GOBIOM, https://gobiomdbplus.com) or focused only on cancer (OncoMX (Dingerdissen, et al., 2020), CIViCmine (Lever, et al., 2019) and ResMarkerDB (Pérez-Granado, et al., 2019)).

# Results

The results of the evaluation of BIONDA and the comparison with MarkerDB and OMIM are shown in supplementary file 2, Table S1, Table S2, Table S3 and Table S4. As can be seen in Table S1 and Table S3, from the 627 correct named entities BIONDA has found 600 (MarkerDB: 268, OMIM: 555) and from the 394 correct relations BIONDA has detected 366 (MarkerDB: 29, OMIM: 75). For BIONDA only 44 false positive named entities and 74 false positive relations were recorded, consequently, BIONDA outperforms MarkerDB and OMIM regarding recall and F1 score (see Table S2 and Table S4). On the other hand, BIONDA is outperformed by MarkerDB and OMIM regarding precision, because no false positives were recorded for them.

**Table S1.** TP, FP and FN named entity recognitions

|  | **BIONDA** | **MarkerDB** | **OMIM** |
| --- | --- | --- | --- |
| **TP** | 600 | 268 | 555 |
| **FP** | 44 | 0 | 0 |
| **FN** | 27 | 316 | 59 |

The numbers of true positive (TP), false positive (FP) and false negative (FN) taggings of named entities that were found in the 200 randomly selected sentences from BIONDA are shown here. Additionally, this is compared with the content of MarkerDB and OMIM.

**Table S2.** Precision, recall and F1 score for named entity recognition

|  | **BIONDA** | **MarkerDB** | **OMIM** |
| --- | --- | --- | --- |
| **Precision** | 0.9317 | 1.0 | 1.0 |
| **Recall** | 0.9569 | 0.4589 | 0.9039 |
| **F1 score** | 0.9441 | 0.6291 | 0.9495 |

Precision, recall and F1 score computed based on the results for named entity recognition summarized in Table S1 are shown.

**Table S3.** TP, FP and FN relation extractions

|  | **BIONDA** | **MarkerDB** | **OMIM** |
| --- | --- | --- | --- |
| **TP** | 366 | 29 | 75 |
| **FP** | 74 | 0 | 0 |
| **FN** | 28 | 356 | 309 |

The numbers of true positive (TP), false positive (FP) and false negative (FN) taggings of relations that were found in the 200 randomly selected sentences from BIONDA are shown here. Additionally, this is compared with the content of MarkerDB and OMIM.

**Table S4.** Precision, recall and F1 score for relation extraction

|  | **BIONDA** | **MarkerDB** | **OMIM** |
| --- | --- | --- | --- |
| **Precision** | 0.8318 | 1.0 | 1.0 |
| **Recall** | 0.9289 | 0.0753 | 0.1953 |
| **F1 score** | 0.8777 | 0.1401 | 0.3268 |

Precision, recall and F1 score computed based on the results for relation extraction summarized in Table S3 are shown.

**References**

Amberger, J.S.*, et al.* OMIM.org: leveraging knowledge across phenotype-gene relationships. *Nucleic Acids Res* 2019;47(D1):D1038-D1043.

Bravo, À.*, et al.* A knowledge-driven approach to extract disease-related biomarkers from the literature. *Biomed Res Int* 2014;2014:253128.

Dingerdissen, H.M.*, et al.* OncoMX: A Knowledgebase for Exploring Cancer Biomarkers in the Context of Related Cancer and Healthy Data. *JCO Clin Cancer Inform* 2020;4:210-220.

Lever, J.*, et al.* Text-mining clinically relevant cancer biomarkers for curation into the CIViC database. *Genome Med* 2019;11(1):78.

Pérez-Granado, J., Piñero, J. and Furlong, L.I. ResMarkerDB: a database of biomarkers of response to antibody therapy in breast and colorectal cancer. *Database (Oxford)* 2019;2019.

Wishart, D.S.*, et al.* MarkerDB: an online database of molecular biomarkers. *Nucleic Acids Res* 2021;49(D1):D1259-D1267.
